# Magnitude and Determinant Factors of Postpartum Depression Among Mothers Attending Their Postnatal and Vaccination Services at Public Health Institutions of Addis Ababa, Ethiopia

**DOI:** 10.3389/fpubh.2022.882205

**Published:** 2022-05-09

**Authors:** Getu Engida Wake, Girma Wogie Fitie, Betelhem Ashenafi, Mesfin Tadese, Saba Desta Tessema

**Affiliations:** Department of Midwifery, Institute of Medicine and Health Science, Debre Berhan University, Debre Berhan, Ethiopia

**Keywords:** depression, Ethiopia, postnatal, postpartum depression, mothers

## Abstract

**Background:**

The postpartum period is known as a high-risk period for the onset of different maternal mental health problems. Globally, 10–20% of postnatal mothers suffer from depressive symptoms. This study aimed to assess the magnitude and determinant factors of postpartum depression among mothers attending their postnatal and vaccination services at public health institutions in Addis Ababa, Ethiopia.

**Methodology:**

Health institution-based cross-sectional study was conducted among 461 postnatal mothers attending public health institutions in Addis Ababa from 15 May 2021 to 15 July 2021. A multistage sampling technique was employed to select the public health institutions and a systematic random sampling method was used to get selected, postnatal mothers. Epidata version 3.1 and SPSS version 25 were used for data entry and analysis, respectively. *P*-value ≤ 0.05 was used as a cut point of statistical significance in multivariable binary logistic regression.

**Results:**

From total postnatal mothers 91(19.7%) of them had postpartum depression. Occupational status [AOR = 3.39, 95% CI: 1.04, 8.15], marital status [AOR = 2.69, 95% CI =1.33, 5.45], income management [AOR = 3.76, 95% CI: 1.53, 8.21], sex of baby [AOR = 5.07, 95% CI: 1.24, 20.69], history of child death [AOR = 6.93, 95% CI: 2.67, 15.79], unplanned pregnancy [AOR = 3.08, 95% CI: 1.65, 7.93], negative life event [AOR = 2.39, 95% CI: 1.03, 5.39], substance use during pregnancy [AOR = 6.23, 95% CI: 2.72, 20.05], history of depression [AOR = 5.08, 95% CI: 1.79, 14.39], and marriage satisfaction [AOR = 6.37, 95% CI: 2.63, 14.29] were determinant factors of postpartum depression.

**Conclusion:**

The prevalence of postpartum depression in this study is high compared to national findings. Occupational status, marital status, income management, sex of baby, history of child death, unplanned pregnancy, negative life event, substance use during pregnancy, history of depression, and marital satisfaction were determinant factors of postpartum depression. The ministry of health should integrate mental health services with existing maternal health care services. It would be better if all healthcare professionals working in the maternal and child health unit will routinely screen postpartum depressive symptoms and link them to mental health services.

## Introduction

The postpartum period is known as a high-risk period for the onset of different mental health problems such as postpartum blue, major depression, and postpartum psychosis (1), and depression is the most periodically happening psychiatric problem among reproductive-age women (2). The American psychiatric association (APA), defined postpartum depression as the occurrence of a major depressive episode (MDE) within 4 weeks after delivery (3) and characterized by loss of interest in usual events, sleep challenges, feelings of sadness, fatigability, problems of appetite, and difficulty in coping with daily activities (4). Globally, 10–20% of postnatal mothers suffered from depressive symptoms during their postpartum period (5). The magnitude of postpartum depression (PPD) raised by 18.4% between 2005 and 2015 years globally (6).

Different kinds of literature had shown that the prevalence of postpartum depression varies globally and it was lower among women from Europe, Australia, and the United States of America (USA) compared to women from Asia, South Africa (7) and it was about 10% in developed countries (6) and nearly 20% in developing countries (8). Evidence indicated that the magnitude of postpartum depression (PPD) in China, Japan, India, and Bangladesh was 6.7, 21, 11–16, and 39.4%, respectively (9–12). The world health organization (WHO) reported, ~20–40% of women in developing countries experienced depressive symptoms during pregnancy or after childbirth (13). African countries constitute a larger burden of postpartum depression (PPD) (14), and the prevalence of postpartum depression was 27.1% in southwestern Uganda (15), 27.5% in Egypt (16), and 35.6% in Nigeria (17). The world health organization report indicated that depression is the leading cause of disease for reproductive age group women (18), and a major public health concern in developing countries currently (6).

Postnatal depression (PND) has several maternal, neonatal, and infant health consequences such as maternal morbidity (19), social problems (20), physical damage (21), suicide (22), infant growth retardation (23), impaired child development (24), behavioral changes (25), and repeated diarrheal disease (26). Factors such as age (27), low household income (28), and preterm/low birth weight infants (29) were determinant factors of postpartum depression. Not only these, but also lack of social support, obstetric complication, previous history of depression, poor marital relationship, and unintended pregnancies also contributing factors to the development of postpartum depression (30–32). According to the report of different studies conducted in different parts of Ethiopia, the magnitude of postpartum depression ranges from 13.11% (33) to 33.82% (34).

Even if the world health organization had launched the mental health gap action program (mhGAP) intended to integrate mental health interventions with the existing maternal health services (14, 35), postpartum depression screening, diagnosis, and referral of clients for appropriate mental health services were often neglected in the health care system of several countries including Ethiopia (36). In addition, about 80% of postnatal mothers with psychological and neurological problems did not access appropriate health care services in Ethiopia and other developing countries (35). In Ethiopia, postnatal health care is majorly concerned with obstetrical/gynecological problems of the mother and newborn health, while the psychosocial well-being of the mother was given little attention even if early identification of mothers at risk of developing postpartum depression will enable us for timely referral, diagnosis, and appropriate management. In addition, little is known about the magnitude of postpartum depression and contributing factors in the current study area, and some previous studies were used a small sample size and didn't consider important potential risk factors. Therefore, this study aimed to assess the magnitude and determinant factors of postpartum depression among postnatal mothers attending their postnatal and vaccination services at public health institutions in Addis Ababa, Ethiopia.

## Methodology

### Study Setting and Period

The study was conducted in Addis Ababa, Ethiopia from May 15/2021 to July 15/2021. There are 10 sub-city and 116 woreda administrations and a total of 12 government-owned hospitals, 98 public health centers, 31 private hospitals, and 700 different level private clinics in Addis Ababa and each sub-city has more than one public health center and hospital. We included all selected public health institutions from three sub-cities (Lideta sub-city, Nifasilik lafto sub-city, and Akaki kality sub-city) in our study.

### Study Participants

There were a total of 937 postnatal mothers who attended all **ten** public health institutions of three sub-cities of Addis Ababa during the data collection period and 461 postnatal women who were selected using proportional to size allocation and attended the selected public health institutions of the three sub-cities of Addis Ababa for postnatal care and vaccination services within 6 weeks after delivery during the study period were include in our study.

### Study Design

Health institution-based cross-sectional study was conducted among three hospitals and seven health centers of the three sub-cities in Addis Ababa.

### Inclusion and Exclusion Criteria

Postnatal women who came for postnatal care and vaccination services within 6 weeks after delivery in selected public health institutions during the data collection period were included, while postnatal mothers who were seriously sick, who could not come to public health institutions, and who were unable to respond during the data collection time were excluded from the study.

### Sample Size Determination, Sampling Technique, and Procedure

The required sample size was determined using single population proportion formula based on the assumptions of a 95% confidence interval, 5% margin of error, and 25% proportion of postpartum depression from the study conducted in Gondar (37).


$$\begin{array}{l} \text{n}=\frac{{\left(\text{Zα}/2\right)}^{2}p\;\left(1-p\right)}{{\text{d}}^{2}} \end{array}$$

where;

*n*: the number of participants to be interviewed,

(*Z* α/2)2: standardized normal distribution value for the 95% CI, = 1.96,

*P*: proportion of postpartum depression (25%) taken from a study conducted in Gondar and *d*: margin of error taken as 5%.


$$\begin{array}{l} n=\frac{{\left(1.96\right)}^{2}\;0.25\left(1-0.25\right)}{0.05^{2}} \end{array}$$

**Figure 1:**
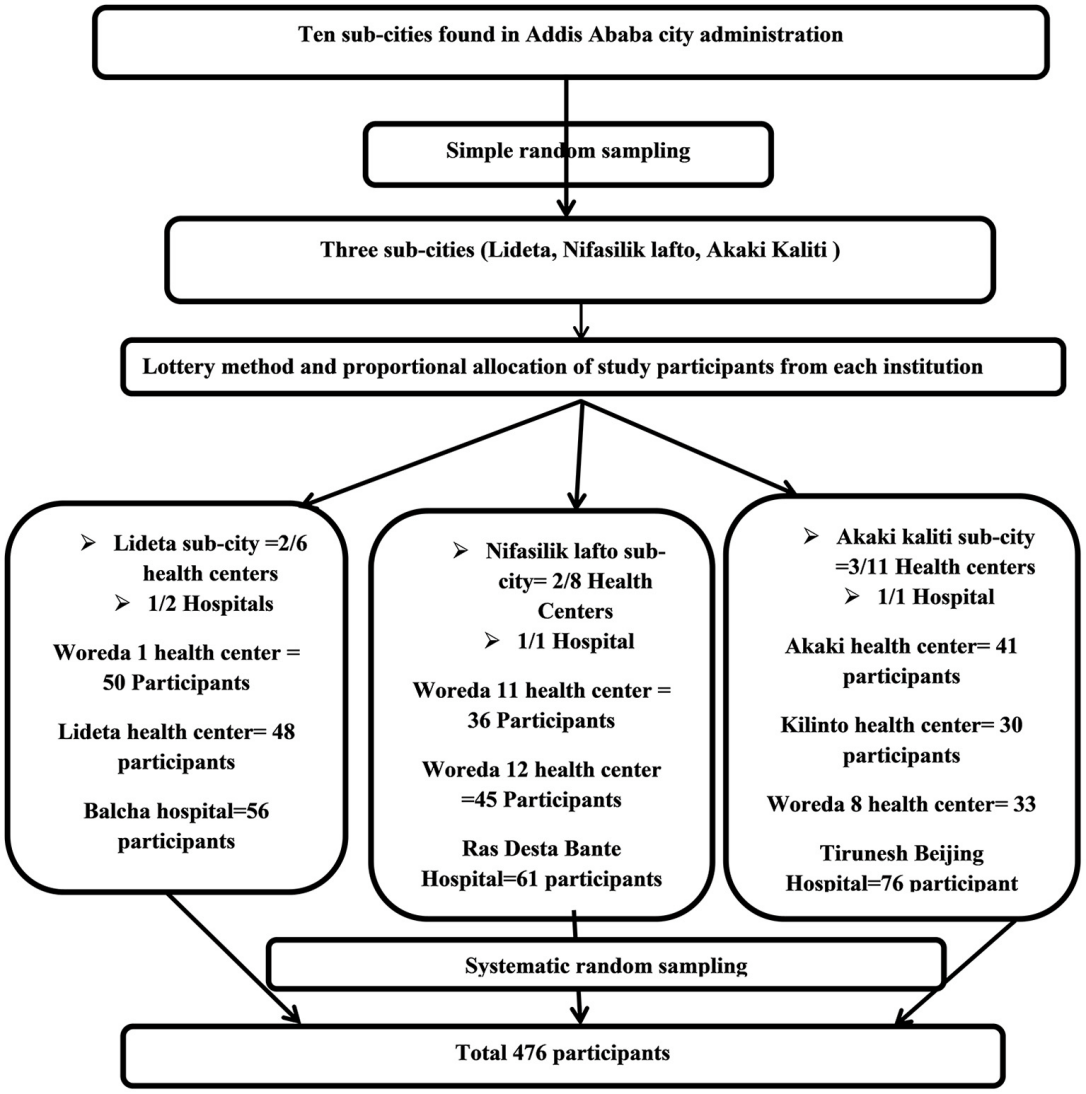
Schematic presentation of sampling procedure of the study.

*n* = 288.12–288, by considering 10% of the non-response rate, the final sample size became 317. Since the multistage sampling technique was used, the sample size was multiplied by the design effect of 1.5, and the final sample size was raised to 476 study participants. A multistage sampling technique was employed to select the public health institutions. In the beginning, out of ten sub-cities found in Addis Ababa city, three sub-cities (Lideta, Nifasilik lafto, and Akaki-Kaliti) were selected using a simple random sampling method. Then, out of a total of 25 health centers found in the three selected sub-cities, a total of 7 health centers (two from Lideta sub-city, two from Nifasilik lafto sub-city, and three from Akaki kaliti sub-city) were selected by a lottery method. Among four hospitals found in the three selected sub-cities, three hospitals (Tirunesh Beijing general hospital from Akaki kaliti sub-city, Ras Desta hospital from Nifasilik lafto sub-city, and Balcha hospital from Lideta sub-city) were selected by simple random sampling. Lastly, ten public health institutions (seven health centers and three hospitals) were included during the data collection period in our study. The numbers of postnatal mothers who visited the public health institutions which were surveyed each health institution were allocated proportionally and the estimation was made depending on the number of postnatal mothers who visited each health institution for the previous 2 months. The proportional allocation was calculated using the following formula:


$$\begin{array}{l} \text{nj}=\text{n}/{\text{N}}^{*}\text{Nj} \end{array}$$

where;

*nj*: sample size of the *j*th health institution,

*n*: total sample size,

*Nj*: number of postnatal mothers who visited the *j*th health institution in the previous 2 months,

*N*: total number of postnatal mothers who visited all public health institutions in the previous 2 months. Finally, Systematic random sampling was used to select postnatal mothers to be included in the study after determining the sampling fraction (*k* = 2th) by dividing the total number of women expected to visit all public health institutions in the previous 2 months by the total number of postnatal mothers to be interviewed and the first participant was determined by lottery method (*K* = 937/476 = 2nd) (Figure 1).

### Study Variables

Postpartum depression was considered as a dependent variable and maternal socio-demographic characteristics(age, educational status, economic status, marital status, and employment), social support status (poor husband support, domestic violence, childbirth without the presence of any relatives, the status of satisfaction with their mother-in-law, the status of satisfaction with their marriage, substance use before and during the current pregnancy, obstetrics factors(parity, unplanned pregnancy, losing or hospitalizing a baby, mode of delivery, pregnancy complication or illness, stressful life event during pregnancy, and sex of the baby), previous psychiatric history (history of depression and family history of psychiatric problems) were independent variables.

### Operational Definitions

Postpartum depression: postpartum depression is a psychiatric disorder that occurs in women following delivery up to 6 weeks. According to Edinburgh postnatal depression scale (EPDS) questions 1, 2, and 4 are scored 0, 1, 2, and 3 with the first choice scored as 0 and the last choice scored as 3. Questions 3, 5–10 are reversely scored, with the first choice scored as 3 and the last choice scored as 0. After adding up all the scores, those women who scored ≥13 thresholds were considered to have postpartum depression (38).

Social support: the perception that one is cared for or has assistance available from other people (39).

Socio-cultural; is a set of beliefs, customs, practices, and behavior that exist in a certain society (40).

*History of depression:* women who had a previous history of mental illness which was characterized by severe feelings of sadness, hopelessness, and loss of interest in activities (41).

### Methods of Data Collection Tool and Quality Control

The structured face-to-face interviewer-administered questionnaire which was adapted from different kinds of literature was used to collect information regarding socio-demographic characteristics, obstetric factors, substance use status, and social support characteristics of postnatal mothers. Besides, the 10 items questionnaire of the Edinburg postnatal depression scale (EPDS) of ≥13 thresholds were adopted and used to assess postnatal depression. This tool was validated as a screening tool to assess postnatal depressive symptoms in Ethiopia among postnatal mothers (38, 42). These questionnaires were designed in the English language, translated into Amharic, and back to the English language for consistency of collected data. In addition, to maintain the quality of data, seven-degree pharmacy and three adult nursing masters were recruited as data collectors and supervisors respectively and they were given training of 2 days about the overall research objective including data collection procedures, tools, and how to fill the data. Besides, the questionnaires were pre-tested in 10% of the sample size in Selam health center 3 weeks before the actual data collection period, and necessary amendments such as language clarity and appropriateness of the tools were done based on the findings of the pretest before the actual data collection time. Collected data was reviewed and checked for completeness and consistency by supervisors and the principal investigator daily.

### Data Processing and Analysis

Finally, the collected data were cleaned, coded, and entered into Epidata version 3.1 and exported to the statistical package for social science (SPSS) version 25 for analysis. Descriptive statistics like mean, median, standard deviation, and percentage were used to summarize the data. Bivariable and multivariable binary logistic regression analyses were used to identify the determinant factors of postnatal depression and *P-*values < 0.2 and 0.05 were considered statistically significant for bivariable and multivariable binary logistic regression, respectively. The overall results were presented in texts, tables, and figures.

## Results

### Socio-Demographic Characteristics

Of a total of 476 study participants, 461 (96.85%) postnatal mothers have participated in the study. Concerning residence and age category, 454 (98.5%) and 299 (64.9%) of the study participants resided in an urban area and age category of 25–34, respectively. Regarding religions and educational status of study participants, 368 (79.8%) and 450 (97.6%) of them were orthodox religion followers and had attended school, respectively. Of the total study participants, 59 (12.8%) of them reported they had worked during the postpartum period and 193 (41.9%) reported they had a monthly family income of >5,001 Ethiopian Birr (Table 1).

**Table 1:** Socio-demographic characteristics of postnatal mothers attending public health institutions in Addis Ababa, Ethiopia, 2021 (*n* = 461).

**Characteristics**	**Frequency**	**Percent**
**Residence**
Urban	454	98.50%
Rural	7	1.50%
**Age in years**
15–24	123	26.70%
25–34	299	64.90%
>35	39	8.50%
**Religion**
Orthodox	368	79.80%
Catholic	26	5.80%
Protestant	45	9.80%
Muslim	20	4.30%
Others	2	0.40%
**Marital Status**
Single	40	8.70%
Married	417	90.50%
Divorced/separated	2	0.40%
Widowed	2	0.40%
**Have you ever attended school?**
Yes	450	97.60%
No	11	2.40%
**Highest level of school you attended**
Primary school	12	2.60%
Secondary school	125	27.80%
Technical/vocational	46	10.20%
Diploma	94	20.90%
Degree and above	173	38.40%
**Occupational status**
Student	41	8.90%
Governmental employee	235	51.00%
Non-governmental employee	30	6.50%
Unemployed	10	2.20%
Merchant	52	11.30%
Housewife	75	16.30%
Others	18	3.90%
**Husband's occupational status**
Student	2	0.50%
Governmental employee	214	51.20%
Non-governmental employee	67	16.00%
Merchant	132	31.60%
Farmer	1	0.20%
Others	2	0.50%
**Do you work during the postpartum period?**
Yes	59	12.80%
No	402	87.20%
**The monthly average income in birr**
<1,500	32	6.90%
1,501–3,000	86	18.70%
3,001–5,000	150	32.50%
>5,001	193	41.90%
**Difficult to manage income**
Yes	51	11.10%
No	410	88.90%

### Obstetrical Characteristics

Among the total study participants, 125 (27.1%) of them reported it was their first pregnancy and 64 (13.9%) of them reported they had a history of abortion. Concerning pregnancy status and method of delivery, 445 (96.5%) and 380 (82.4%) of the respondents planned their last pregnancy and gave birth through the vagina, respectively (Table 2).

**Table 2:** Obstetric characteristics of postnatal mothers attending public health institutions in Addis Ababa, Ethiopia, 2021 (*n* = 461).

**Characteristics**	**Frequency**	**Percent**
**Number of pregnancy**
1	125	27.10%
02-Mar	316	68.50%
>4	20	4.30%
**Have you ever had an abortion?**
Yes	64	13.90%
No	397	86.10%
**Experienced death of baby**
Yes	20	4.30%
No	441	95.70%
**Any children hospitalized**
Yes	15	3.30%
No	446	96.70%
**Was your last pregnancy planned?**
Yes	445	96.50%
No	16	3.50%
**Illness/complication during last pregnancy**
Yes	28	6.10%
No	433	93.90%
**Presence of ANC visit**
Yes	461	100%
No	0	0%
**Number of ANC visits**
<4	9	2%
Grater or equal to 4	452	98%
**Place of delivery**
Home	0	0%
Health Institution	461	100%
**Mode of delivery**
Vaginal	380	82.40%
Cesarean section	44	9.50%
Instrumental delivery	37	8.00%
**Status of the last infant at birth**
Alive	449	97.40%
Dead	12	2.60%
**Gestational age of last pregnancy**
Term	443	96.10%
Preterm	18	3.90%
**Weight of the last infant**
Normal birth weight	432	93.70%
Low birth weight	29	6.30%
**The onset of labor for the last infant**
Spontaneous	380	82.40%
Induced	81	17.60%
**Sex of your last baby**
Male	202	43.80%
Female	259	56.20%
**Sex of the last baby**
Desired	441	95.70%
Undesired	20	4,3%
**Negative life events during pregnancy**
Yes	13	2.80%
No	448	97.20%

### Substance Use, Previous Psychiatric History, and Social Support

Concerning substance use, 436 (94.6%) of study participants reported the absence of substance use before this pregnancy, and 454 (98.5%) of postnatal women reported the absence of substance use during their last pregnancy. Regarding, previous psychiatric history, 6 (10%) of study participants had a previous history of postpartum depression and 31 (6.7%) of the respondents had reported the presence of a family history of mental illnesses. About 381 (92.5%) of the study participants were satisfied with their marriage (Table 3).

**Table 3:** Social support of postnatal mothers attending public health institutions in Addis Ababa, Ethiopia, 2021 (*n* = 461).

**Characteristics**	**Frequency**	**Percent**
**Ever experienced abuse at home**
Yes	14	3.00%
No	447	97.00%
**Marriage satisfaction**
Yes	381	92.50%
No	31	7.50%
**Support from the father of a child**
Yes	440	96.30%
No	17	3.70%
**Relatives present in the birthplace of the last baby**
Yes	444	96.30%
No	17	3.70%
**Satisfied by a relationship with in-laws**
Yes	408	96.00%
No	17	4.00%

### Prevalence of Postpartum Depression

The prevalence of depressed and non-depressed among postnatal mothers were 91[19.7%, (95% CI = 16.1, 23.4)] and 370[80.3% (95% CI = 76.6, 83.9)], respectively.

### Factors Associated With Postpartum Depression Among Postnatal Mothers

Variables such as marital status, attendance of school, occupational status, difficulty managing income, history of abortion, history of child death, history of children being hospitalized, unplanned pregnancy, illness/complication during pregnancy, the status of the last infant, weight of the infant, the onset of labor, sex of baby, negative life events, history substance use before pregnancy, substance use during pregnancy, family history of mental illnesses, history of depression, abuse at home, satisfaction with marriage, support from baby father and status of satisfaction with mother-in-law were significantly associated with maternal postpartum depression in bivariable binary logistic regression. But, multivariable binary logistic regression analysis results indicated variables such as occupational status, marital status, income management, sex of baby, history of child death, unplanned pregnancy, negative life events, substance use during pregnancy, history of depression, and marital satisfaction were significantly associated with maternal postpartum depression development.

Accordingly, unemployed study participants were 3.39 times more likely to develop postpartum depression than those who were employed [AOR = 3.39, 95% CI: 1.04, 8.15]. Unmarried postnatal mothers were 2.69 times more likely to develop postpartum depression than those who were married [AOR = 2.69, 95% CI =1.33, 5.45]. Respondents who had a problem in managing their income were 3.75 times more likely to develop postpartum depression than those who had no problem in managing their income [AOR = 3.75, 95% CI: 1.53, 8.21]. Postnatal mothers who delivered a baby with undesired sex were 5.07 times more likely to have postpartum depression than those who delivered a baby with desired sex [AOR = 5.07, 95% CI: 1.24, 20.69]. Study participants who had a history of child death were 6.93 times more likely to develop postpartum depression than those who had no history of child death [AOR = 6.93, 95% CI: 2.67, 15.79]. Study respondents who had not planned their last pregnancy were 3.08 times more likely to experience postpartum depression as compared to their counterparts [AOR = 3.08, 95% CI: 1.65, 7.93]. Postnatal mothers who faced negative life events during pregnancy were 2.39 times more likely to have postpartum depression than their counterparts [AOR = 2.39, 95% CI: 1.04, 5.39]. Postnatal mothers who had used substances during pregnancy were 6.23 times as compared to their counterparts [AOR = 6.23, 95% CI: 2.72, 20.05]. Study participants who had a history of depression were 5.08 times more likely to develop postpartum depression compared to their counterparts [AOR = 5.08, 95% CI: 1.79, 14.39]. Postnatal mothers who were not satisfied with their marriage were 6.37 times more likely to develop postpartum depression than their counterparts [AOR = 6.37, 95% CI: 2.63, 14.29] (Table 4).

**Table 4:** Bivariable and multivariable binary logistic regression analysis for factors associated with postpartum depression among postnatal mothers attending public health institutions in Addis Ababa, Ethiopia, 2021 (*n* = 461).

**Variables**	**Depressed**	**Not depressed**	**COR (95% C.I)**	**AOR (95% C I)**	***P*-value**
**Attended school**					
Yes	84	366	1	1	
No	7	4	7.63 (2.18, 26.64)	5.66 (0.99, 32.45)	0.052
**Occupational status**					
Employed	83	368	1	1	
Unemployed	8	2	**17.74 (3.69, 85.05)**	**3.39 (1.04, 8.15)**	**<0.001**
**Marital status**					
Married	75	346	1	1	
Unmarried	16	24	**3.08 (1.56, 6.07)**	**2.69 (1.33, 5.45)**	**0.006**
**Difficult to manage income**					
Yes	37	14	**17.42 (8.84, 34.33)**	**3.75 (1.53, 8.21)**	**<0.001**
No	54	356	1	1	
**Desired sex for the last baby**					
Desired	78	363	1	1	
Undesired	13	7	**8.64 (3.34, 22.37)**	**5.07 (1.24, 20.69)**	**0.024**
**Ever had an abortion?**					
Yes	21	43	2.28 (1.28, 4.08)	1.66 (0.57, 4.81)	0.234
No	70	327	1	1	
**Ever experienced the death of baby**					
Yes					
No	15	5	**14.41 (5.08, 40.84)**	**6.93 (2.67, 15.79)**	**0.010**
	76	365	1	1	
**Any children hospitalized**					
Yes	10	5	9.01 (2.99, 27.08)	0.34 (0.01, 9.54)	0.527
No	81	365	1	1	
**Was your last pregnancy planned?**					
Yes	78	367	1	1	
No	13	3	**20.39 (5.68, 73.25)**	**3.08 (1.65, 7.93)**	**<0.001**
**Illness/complication during last pregnancy**					
Yes	11	17	2.86 (1.29, 6.33)	1.19 (0.33, 4.31)	0.832
No	80	353	1	1	
**Weight of the last infant**					
Normal birth weight	82	350	1	1	
Underweight	9	20	1.92 (0.84, 4.37)	0.21 (0.05-0.96)	0.054
**Status of the last infant at birth**					
Alive	84	365	1	1	
Dead	7	5	6.08 (1.88, 19.64)	1.93 (0.16-23.37)	0.607
**The onset of labor for the last infant**					
Spontaneous	68	312	1	1	
Induced	23	58	1.82 (1.05, 3.15)	1.77 (0.64, 4.91)	0.272
**Negative life event during your last pregnancy**					
Yes	9	4	**10.04 (3.02, 33.41)**	**2.39 (1.04, 5.39)**	**0.006**
No	82	366	1	1	
**Substance use before pregnancy**					
Yes	14	11	**5.93 (2.59, 13.57)**	**0.43 (0.09, 2.02)**	**0.283**
No	77	359	1	1	
**Substance use during pregnancy**					
Yes	6	1	**26.05 (3.09, 219.20)**	**6.23 (2.72, 20.05)**	<**0.001**
No	85	369	1	1	
**Relative suffered from mental illness**					
Yes	13	18	3.26 (1.53, 6.93)	0.44 (0.10, 1.89)	0.268
No	78	352	1	1	
**Previous history of depression**					
Yes	27	19	**7.79 (4.09, 14.85)**	**5.08 (1.79, 14.39)**	**0.002**
No	64	351	1	1	
**Experienced any abuse in your home**					
Yes	8	6	5.85 (1.98, 17.31)	4.15 (0.78, 21.96)	0.095
No	83	364	1	1	
**Satisfied with marriage**					
Yes	49	332	1	1	
No	22	9	**16.56 (7.21, 38.04)**	**6.37 (2.63, 14.29)**	**<0.001**
**Father of your child's support**					
Yes	79	361	1	1	
No	9	8	5.14 (1.92, 13.74)	2.70 (0.28-26.16)	0.493
**Satisfied by the relationship with mother-in-law**					
Yes	65	343	1	1	
No	9	8	5.94 (2.21, 15.95)	1.01 (0.10, 10.21)	0.525

*P-value is significant at P < 0.05, 1: reference, AOR, adjusted odds ratio; CI, confidence interval; COR, crude odds ratio. Bold value indicates significant association between independent variables and dependent variable*.

## Discussion

This study aimed to assess the magnitude and determinant factors of postpartum depression among mothers attending their postnatal and vaccination services at public health institutions in Addis Ababa, Ethiopia. The study revealed that the prevalence of postpartum depression (PPD) was 19.7%. This finding was in line with the result of a study conducted in Nekemte town, Ethiopia 20.9 % (43), and Saudi Arabia 20.9 % (44). But higher than the result of the study conducted in Eritrea 7.4 % (45), and Sudan 10.9 % (46). This difference might be related to methodological differences such as sample size, sampling procedure, the timing of the postpartum period, assessment tool rating scale difference. The study conducted in Eritrea used the standard diagnostic and statistical manual of mental disorders fifth edition for diagnosis of postpartum depression (45), while the study conducted in Sudan (46) used Edinburg postnatal depression scale (EPDS) with >12 cuts of point for diagnosis of postpartum depression.

In addition, this result was higher than the results of three studies conducted in different parts of Ethiopia such as Harar (33), Debre Berhan (47), and eastern Ethiopia (48) which reported the prevalence of PPD as 13.11, 15.6, and 16.3%, respectively. Moreover, the prevalence of postpartum depression in this study was lower than the result of a study conducted in southwestern Uganda 27.1% (15), Egypt 27.5% (16), Pakistan 31% (49), Vientiane Capital 31.8% (41), Nigeria 35.6% (17), and Bangladesh 39.4% (12). This variation might be associated with socio-cultural differences, assessment tools, the difference in the cut of point of EPDS, the difference in the sampling procedure, and included study participants. The study conducted in southwestern Uganda (15) used the diagnostic and statistical manual of mental disorders V for diagnosis of postpartum depression while Edinburg postnatal depression scale (EPDS) was used in our study. Edinburg postnatal depression scale (EPDS) with ≥10 thresholds were used in a study conducted in Bangladesh (12) and Vientiane Capital (41), while minimum Edinburg postnatal depression scale (EPDS) ≥13 thresholds were considered for the diagnosis of postpartum depression in our study. Besides, the prevalence of postpartum depression (PPD) in this study was lower than the report of three studies conducted in different regions of Ethiopia such as Addis Ababa (50), Gondar (37), and southeast Ethiopia (34) showing the prevalence of postpartum depression (PPD) 23, 25, and 33%, respectively. This discrepancy might be associated with the methodological and socio-demographic differences of included study participants.

Unemployed postnatal mothers were over 3 times more likely to develop postpartum depression compared to employed postnatal mothers. This finding was in line with the result of a study conducted in Sudan, Qatar, Lebanon, and the USA (46, 51–53). This could be related to the lack of adequate income among unemployed postnatal mothers which could be the driving cause for the onset of a different psychosocial stressor and postpartum depression. Again, delivery and postpartum periods are known as stressful moments, and unemployment is the main relapsing factor of previous stress, exposing unemployed postnatal mothers to develop postpartum depression (54). But, this finding was different from the result of the study conducted in Eritrea 2020 (45), which indicated that housewives were less likely to develop postpartum depression compared to those employed postnatal mothers.

Those unmarried postnatal mothers were almost 3 times more likely to develop postpartum depression. This finding was in line with the result of the study conducted in Addis Ababa Ethiopia (55), and Uganda (56). Pregnancy and delivery in the absence of marriage have stigma and discrimination in the community of most African countries. Handling these stressful events alone and the absence of support those postnatal mothers could have gotten from their partners might initiate the development of postpartum depression (PPD). Besides, evidence indicated that lack of partner support was a risk factor for the development of postpartum depression (45). Postnatal mothers who had difficulty in managing their income were almost 4 times more likely to have postpartum depression than those who had no problems managing their income. This result was in line with the results of two studies conducted in Ethiopia [Addis Ababa (55) and at the national level in Ethiopia (57)], Eritrea (45), Qatar (51), Taiwan (58), and western Iran (59). The possible explanation is that those postnatal mothers who were of low economic status or had a problem managing their finance might have faced the inability to fulfill all the necessary needs for raising their newborns, which might have caused postpartum depression.

Postnatal mothers who delivered a baby with undesired sex were about 5 times more likely to have postpartum depression than their counterparts. This finding was consistent with the report of the study conducted in Harar, Ethiopia (33) and Uganda (56). Probably, the absence of support from the family members for postnatal mothers because of the delivery of a baby with undesired sex, especially in a family where there were sex preferences, might have stressed the mother to develop postpartum depression. Study participants who had a history of child death were seven times more likely to develop postpartum depression compared to their counterparts. This finding was in line with the results of studies conducted in Mizan-Tepi University teaching hospital, Ethiopia (34) and a systematic review and meta-analysis, Ethiopia (60). The loss of an infant has several negative impacts on maternal life, and mothers who had lost a child before could fear they might lose their newborn as well again, which might have stressed the mother for the development of postpartum depression. Besides, unplanned pregnancy increased the odds of postpartum depression about 3 times. This finding was in line with the reports of two systematic reviews and meta-analyses in Ethiopia (57, 60), Bangladesh (12), and developing countries (61). Probably, those postnatal mothers with unplanned pregnancies might have not fully prepared for pregnancy, childbirth, and child nursing events, which might have stressed the postnatal mothers in turn to develop postpartum depression.

Those postnatal mothers who have faced negative life events during their pregnancy were over 2 times more likely to develop postpartum depression. This finding was similar to the results of the study conducted in Bahir dar town, Ethiopia (62), Egypt (16), Qatar (51), and Saudi Arabia (44). Stressful life events were the major risk factors for the onset of different psychosocial stressors and literature has indicated that exposure to negative life events can cause postpartum depression (63–65). Substance utilization during pregnancy was about six times risky for the development of postpartum depression. This finding was similar to the reports of studies conducted in Addis Ababa, Ethiopia (55), Mizan Aman town, Ethiopia (66), and Nekemte town, Ethiopia (43). According to some evidence, postpartum depressive symptoms were prevalent among postpartum substance users (67) and those with a substance utilization history (29).

Those postnatal mothers who had a previous history of depression were 5 times more likely to develop postpartum depression (PPD) than those who did not have a previous history of depression. This finding was consistent with the results of the study conducted in Egypt (16), Saudi Arabia (44), systematic review and meta-analysis in Ethiopia (60), and the study conducted in developing countries (61). Most of the risk factors for the development of disease either mental or non-mental are recurrent and hormonal imbalance during pregnancy and postpartum might have caused the relapse of the previous depression. Lastly, those study participants who were unsatisfied with their marriage were over 6 times more likely to develop postpartum depression compared to their counterparts. This finding was in line with the results of a systematic review and meta-analysis in Ethiopia (60), Mizan Aman town, Ethiopia (66), and a study conducted in developing countries (61). Probably, this was related to the absence of sharing of burdens such as child caring, household activities, and social responsibilities among couples with poor marital relationships, which could have stressed those postnatal mothers.

## Limitations

Our study was carried out during the global pandemic of COVID-19, which might have increased the prevalence of postpartum depression in Ethiopia. Data about the risk factors of PPD were collected from postnatal women's recall, which leads to under or over-reporting of symptoms of depression. Kansas Marital Satisfaction Scale and 3-item Oslo Social Support Scale were not used to assess the level of marital satisfaction and level of social support, respectively. The cause and effect relationship of the independent with dependent variable was not determined because of the cross-sectional nature of the design. The correlates of postpartum depression explored in this study were not exhaustive and the use of objective measures to assess some of the plausible correlates of postpartum depression was lacking.

## Conclusions

The prevalence of postpartum depression in our study is high and it was an alarming finding which necessitates more attention to be given to maternal mental health problems during the postpartum period. Occupational status, marital status, income management, sex of baby, history of child death, unplanned pregnancy, negative life event, substance use during pregnancy, history of depression, and marital satisfaction were determinant factors of postpartum depression. The ministry of health should integrate mental health services with existing maternal health care services. It would be better if all healthcare professionals working in the maternal and child health care unit will routinely screen postpartum depressive symptoms and link them to mental health services giving special consideration to those postnatal mothers who were unemployed, unmarried, unsatisfied with their marriage, and for those postnatal mothers who had low monthly family income/problem of finance management, undesired sex of a baby, history of child death, unplanned pregnancy, negative life event during last pregnancy, substance use during their pregnancy and previous history of mental health problems.

## Data Availability Statement

All data sets used for this study are available from the corresponding author on request.

## Author Contributions

GW, GF, and BA: conceptualization. MT and ST: methodology. GF, BA, and ST: software and supervision. GW, MT, ST, and BA: formal analysis. GF, MT, and ST: data curation. GW and MT: writing—original draft preparation. GW, GF, BA, MT, and ST: writing—review and editing. GW: visualization. GW, GF, and MT: funding acquisition. All authors have read and approved the final version of the manuscript to be published.

## Funding

Debre Berhan University had covered the costs for data collection instruments, data collectors, and supervisors. Ethical clearance was obtained from the Institutional Health Research Review Committee Board (Ref. No. IHRRCB-019/04/2020) of the Institute of Medicine and Health Science College of Debre Berhan University. But the funder had no role in the decision to publish.

## Conflict of Interest

The authors declare that the research was conducted in the absence of any commercial or financial relationships that could be construed as a potential conflict of interest.

## Publisher's Note

All claims expressed in this article are solely those of the authors and do not necessarily represent those of their affiliated organizations, or those of the publisher, the editors and the reviewers. Any product that may be evaluated in this article, or claim that may be made by its manufacturer, is not guaranteed or endorsed by the publisher.

## Abbreviations

APA: American psychiatric association
AOR: adjusted odds ratio
CI: confidence interval
COR: crudes odds ratio
DMS IV: diagnostic and statistical manual of mental health disorder
EPDS: Edinburgh postnatal depression scale
MCH: maternal and child health
mhGAP: mental health gap action program
MDE: major depressive episode
PNC: postnatal care
PPD: postpartum depression
SPSS: statistical package for social science
USA: United States of America
WHO: world health organization.
